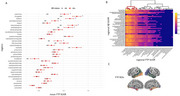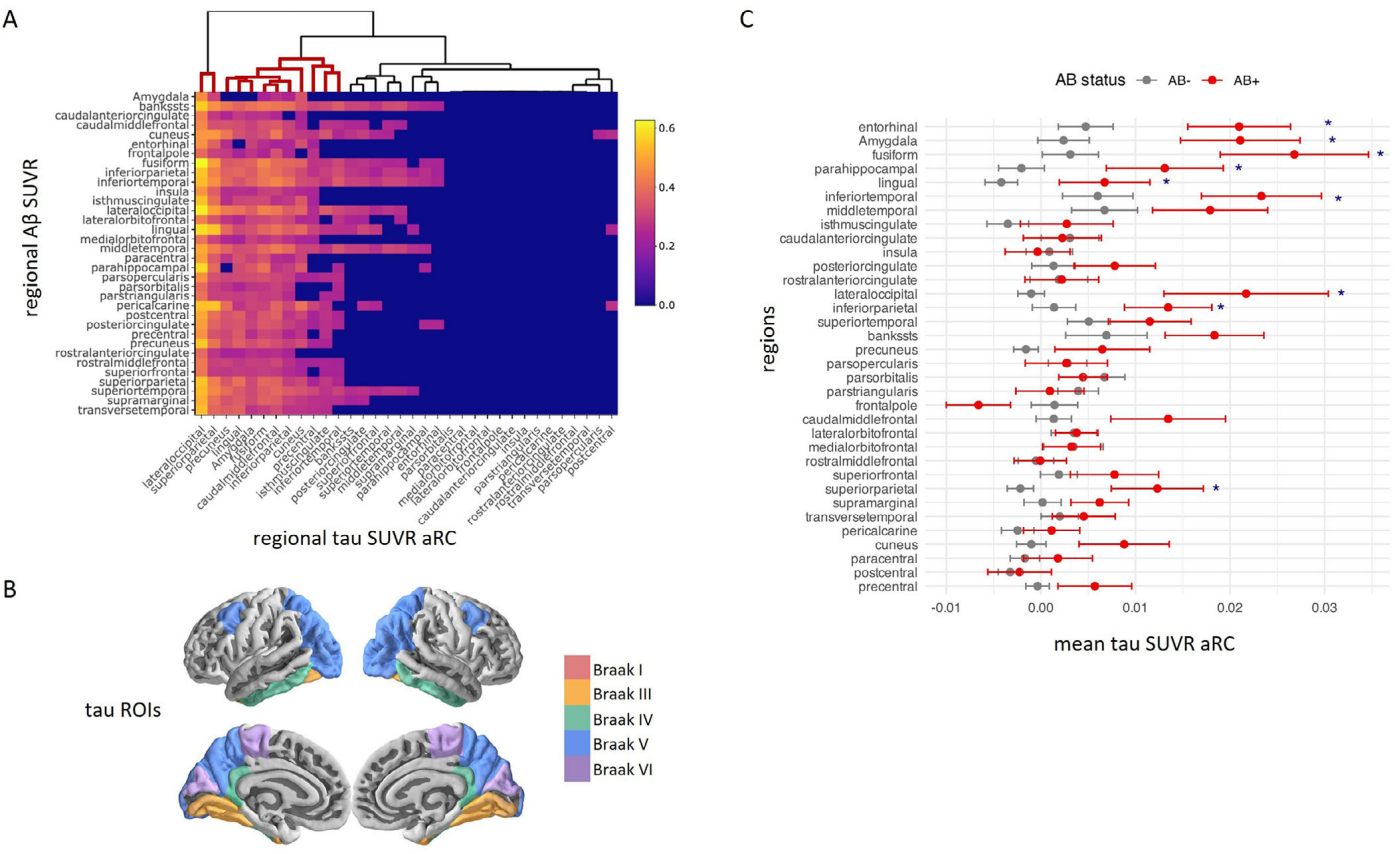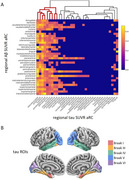# Spatiotemporal associations between amyloid and tau pathologies in preclinical Alzheimer’s disease

**DOI:** 10.1002/alz70861_108933

**Published:** 2025-12-23

**Authors:** Alfonso Fajardo, Ting Qiu, Sylvia Villeneuve

**Affiliations:** ^1^ Douglas Mental Health University Institute, Centre for Studies on the Prevention of Alzheimer's Disease (StoP‐AD), Montréal, QC Canada; ^2^ Integrated Program in Neurosciences, McGill University, Montréal, QC Canada; ^3^ Centre for Studies on Prevention of Alzheimer's disease (StoP‐AD Centre), Douglas Mental Health Institute, Montreal, QC Canada; ^4^ The Douglas Mental Health Institute, Montreal, QC Canada; ^5^ StoP‐AD Centre, Douglas Mental Health Institute Research Centre, Montreal, QC Canada; ^6^ Department of Psychiatry, McGill University, Montréal, QC Canada; ^7^ Douglas Research Centre, McGill University, Montreal, QC Canada

## Abstract

**Background:**

In Alzheimer’s disease (AD), the Thal and Braak staging framework describes the typical regional progression of amyloid‐beta (Aβ) and tau, respectively. However, the spatiotemporal relationships between Aβ and tau remain poorly understood. These interactions may influence the rate and pattern of disease progression. We aimed to a) characterize the regional cross‐sectional associations between Aβ and tau PET signals, b) examine whether regional Aβ accumulation at baseline predicts subsequent regional tau accumulation over time, and c) examine the regional longitudinal associations between Aβ and tau PET signals.

**Method:**

We analyzed baseline (*n* =252) and ∼4.5‐year follow‐up (*n* =108) Aβ‐ and tau‐PET scans from cognitively unimpaired individuals with a family history of AD in the PREVENT‐AD cohort. Regional SUVRs for Aβ and tau were extracted from 34 bilateral regions of interest using the Desikan‐Killiany parcellation. For aim a, region‐wise Pearson correlations were computed between baseline Aβ and tau SUVRs. Non‐significant correlations were thresholded. Hierarchical clustering identified regional groups with similar Aβ–tau associations. For aim b, the same procedure was used for annual rate of change (aRC). We also assessed baseline Aβ vs tau aRC and vice versa. In both analyses, tau SUVRs or aRC were compared between Aβ‐ and Aβ+ groups (Centiloid > 20).

**Result:**

Cross‐sectional analyses showed that Aβ SUVRs across most regions were positively correlated with tau SUVRs in the amygdala and medial/inferior temporal lobes. Similar patterns were observed longitudinally. Baseline Aβ in widespread cortical regions was associated with greater tau aRC in the amygdala, precuneus/posterior cingulum, superior‐parietal, and lateral occipital cortices. In both analyses, Aβ+ individuals showed higher tau deposition in Braak I and Braak III regions.

**Conclusion:**

Beyond the inferior/medial temporal lobe regions commonly included in the tau “temporal meta‐ROI,” we observed significant Aβ associations with both tau and tau aRC in the latero‐occipital and superior‐parietal cortices. Longitudinal analyses further revealed a strong Aβ–tau relationship in the precuneus/posterior cingulum. As these areas correspond to later Braak stages and are not typically emphasized in early AD assessments, their involvement may reflect spatial tau progression and highlight the need to consider them in future studies of preclinical AD.